# Tailoring tumor-recognizable hyaluronic acid–lipid conjugates to enhance anticancer efficacies of surface-engineered natural killer cells

**DOI:** 10.1186/s40580-023-00406-1

**Published:** 2023-12-14

**Authors:** Chae Eun Lee, Sungjun Kim, Hee Won Park, Wonjeong Lee, Ashok Kumar Jangid, Yonghyun Choi, Woo-Jin Jeong, Kyobum Kim

**Affiliations:** 1 Department of Chemical & Biochemical Engineering, Dongguk University, Seoul, 04620 Republic of Korea; 2 Department of Biological Engineering, Inha University, Incheon, 22212 Republic of Korea; 3 Department of Chemical Science and Engineering, Tokyo Institute of Technology, Kanagawa, 226-8501 Japan

**Keywords:** Natural killer cell, Ex vivo cell surface engineering, Hyaluronic acid–Lipid conjugate, Lipid anchor, Hydrophobic membrane interaction

## Abstract

Natural killer (NK) cells have clinical advantages in adoptive cell therapy owing to their inherent anticancer efficacy and their ability to identify and eliminate malignant tumors. However, insufficient cancer-targeting ligands on NK cell surfaces often inhibit their immunotherapeutic performance, especially in immunosuppressive tumor microenvironment. To facilitate tumor recognition and subsequent anticancer function of NK cells, we developed hyaluronic acid (HA, ligands to target CD44 overexpressed onto cancer cells)-poly(ethylene glycol) (PEG, cytoplasmic penetration blocker)-Lipid (molecular anchor for NK cell membrane decoration through hydrophobic interaction) conjugates for biomaterial-mediated ex vivo NK cell surface engineering. Among these major compartments (i.e., Lipid, PEG and HA), optimization of lipid anchors (in terms of chemical structure and intrinsic amphiphilicity) is the most important design parameter to modulate hydrophobic interaction with dynamic NK cell membranes. Here, three different lipid types including 1,2-dimyristoyl-sn-glycero-3-phosphati-dylethanolamine (C14:0), 1,2-distearoyl-sn-glycero-3-phosphatidylethanolamine (DSPE, C18:0), and cholesterol were evaluated to maximize membrane coating efficacy and associated anticancer performance of surface-engineered NK cells (HALipid-NK cells). Our results demonstrated that NK cells coated with HA-PEG-DSPE conjugates exhibited significantly enhanced anticancer efficacies toward MDA-MB-231 breast cancer cells without an off-target effect on human fibroblasts specifically via increased NK cell membrane coating efficacy and prolonged surface duration of HA onto NK cell surfaces, thereby improving HA-CD44 recognition. These results suggest that our HALipid-NK cells with tumor-recognizable HA-PEG-DSPE conjugates could be further utilized in various cancer immunotherapies.

**Graphical Abstract:**

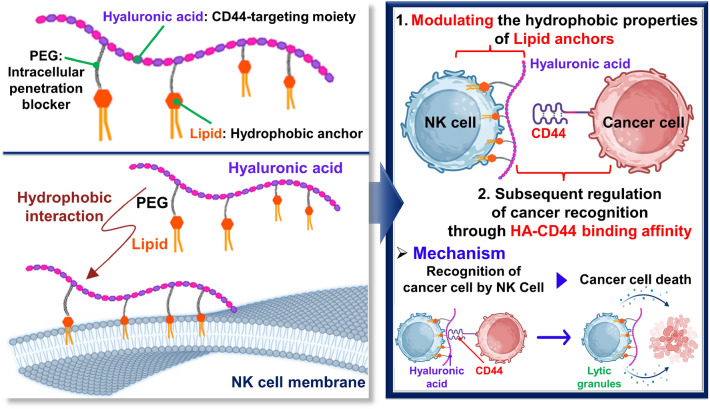

**Supplementary Information:**

The online version contains supplementary material available at 10.1186/s40580-023-00406-1.

## Introduction

Adoptive cell therapy is one novel cancer immunotherapy that can treat malignancies, especially when activated immune cells are used in ex vivo conditions [1, 2]. Among diverse cell types, natural killer (NK) cells stand out as a remarkable population of innate immune cells due to their intrinsic capacities for tumor immune surveillance and robust antitumor responses [3]. Compared to T cells, NK cells exhibit potent cytotoxicity without triggering inflammatory cytokine syndrome [4, 5]. However, inhibitory signals of NK cells prevalent in the tumor microenvironment and less antigen receptors often lead to immune escape, subsequently decreasing their anticancer efficacy [6, 7]. Cell surface engineering could be achieved by various techniques: (1) incorporating lipophilic/hydrophobic molecules that interact with cellular membranes through hydrophobic interactions, (2) covalent modification between proteins and chemical residues on the cell surface, (3) metabolic engineering that intentionally modifies cellular metabolic pathways to generate desired surface compounds, (4) electrostatically functionalizing with cationic polymers on anionic charged cell surfaces. However, chemical-based modifications may disturb the native properties of cells, potentially leading to the unexpected loss of surface protein bioactivity and subsequent effects on cell bioactivity [8, 9]. For example, the development of chimeric antigen receptor (CAR) has been focused on augmenting efficient cancer recognition of immune cells. Nevertheless, such genetic modification shows several drawbacks, including a time-consuming process, unexpected tumorigenesis due to the use of viral vectors (i.e., lentivirus, adenovirus), and challenges in eliminating or deactivating post-administration of CAR cells [10–12].

To overcome these limitations, biomaterial-mediated ex vivo cell surface engineering with polymeric compounds without any genetic modulation of transplantable immune cells has emerged [13, 14]. Among various biomaterials, lipid-mediated polymer conjugates could homogeneously decorate NK cell surfaces through hydrophobic interactions between lipids and NK cell membranes. This in situ cell coating enables sufficient presentation of specific signaling modulators without genetic modification and produces high yields of surface-modified immune cells [11, 15]. On the other hand, the conventional layer-by-layer deposition method typically leads to cation-mediated cytotoxicity and random coverage of cell surfaces [16, 17]. This could result in unexpected interference with signaling receptors and ligands, and inhibit cytokine secretion, ultimately diminishing the efficacy of immune cells [13]. Therefore, lipid-based NK cell surface modification has the potential to compensate for the shortcomings of existing methods.

The most important parameter in ex vivo NK cell surface engineering via lipid conjugates is stable immobilization of materials without penetration into cytoplasm for enhanced specific targeting ability toward cancer cells. Design criteria for optimized lipid conjugates are: (1) minimized endocytosis of surface-presentable materials, (2) non-intervention of intrinsic signaling receptors/ligands of NK cells after coating processes, and (3) durable decoration on NK cell surface against mechanical stress during in vivo infusion and following blood circulation [8]. Moreover, anticancer efficacy of engineered NK cells mediated by materials presented on cell surfaces (target recognition and killing) could vary depending on the size, type, and properties of materials within dynamic and intricate cellular membrane structures [18, 19]. Therefore, it is important to optimize lipid type by considering hydrophobic/hydrophilic balances between lipid conjugates and NK cell membranes, and by modulating lipid structures according to the number of carbons [20, 21].

**Scheme 1:**
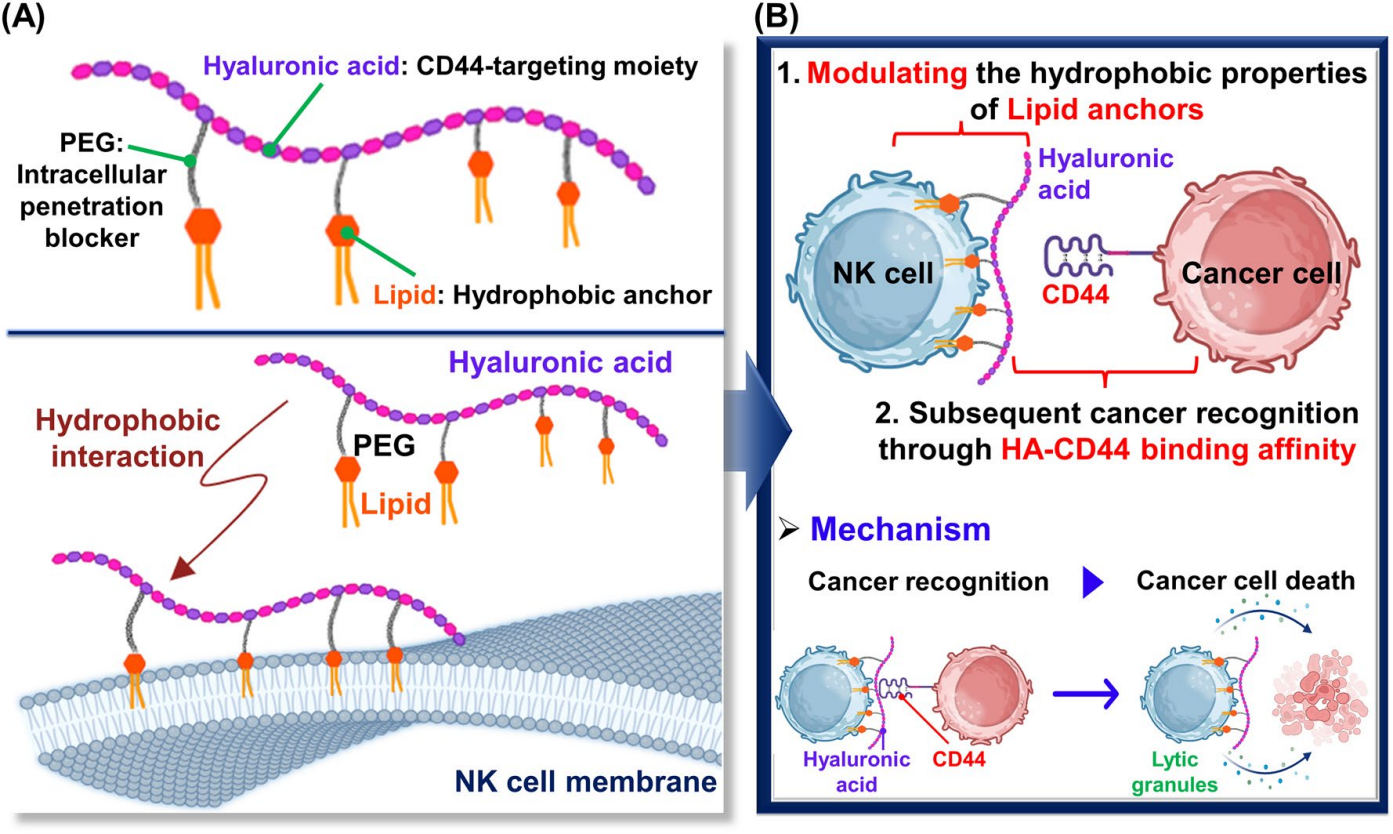
Schematic illustration of HALipid-NK cells to enhance cancer-killing efficacy. **A** Graphical depiction of HA-PEG-Lipid conjugate anchoring to NK cell membrane via hydrophobic interaction. **B** Illustration of cancer recognition and killing strategy by HALipid-NK cells

To this end, we investigated effects of various lipid types on anticancer efficacies of surface-engineered NK cells (HALipid-NK cells). Our lipid conjugates have three major compartments: (1) lipid moiety for anchoring into NK cell membranes via hydrophobic interactions, (2) poly(ethylene glycol) (PEG) acting as an intracellular penetration blocker, and (3) hyaluronic acid (HA) for targeting CD44 overexpressed on aggressive cancer cells (especially triple-negative breast cancer cells) (Scheme 1A). To modulate the hydrophobicity of lipid anchors, 1,2-dimyristoyl-sn-glycero-3-phosphati-dylethanolamine (DMPE, C14:0), 1,2-distearoyl-sn-glycero-3-phosphatidylethanolamine (DSPE, C18:0), and cholesterol (Chol) were selected as lipid candidates. Especially, the two-tail lipids, DMPE and DSPE, were chosen due to their significantly enhanced levels of membrane insertion efficacy when compared to single-tail lipids [22]. Each lipid module was conjugated on a HA backbone with PEG linker as a form of HA-PEG-Lipid. A sequential process for the recognition of target cancer cells and augmentation of cancer-killing efficacy of HALipid-NK cells could be achieved through HA-CD44 binding affinity (Scheme 1B).

## Methods/experimental

### Materials and chemicals

HA was purchased from Lifecore Biomedical, Inc. 1,2-distearoyl-sn-glycero-3-phospho-ethanolamine-N-[amino(polyethylene glycol)-2000] was bought from Nanosoft Polymers (DSPE-PEG-NH_2_). 1,2-dimyristoyl-sn-glycero-3-phosphati-dylethanolamine-N-[amino(polythylene glycol)-2000] (DMPE-PEG-NH_2_) and cholesterol-*N*-[amino(polyethylene glycol)-2000] (Chol-PEG-NH_2_) were obtained from Biopharma PEG Scientific Inc. Dimethylformamide (DMF) (anhydrous), 1-ethyl-3-(3-dimethylaminopropyl) carbodiimide (EDC), N-hydroxy succinimide (NHS), and 4-dimethyl amino pyridine (DMAP) were obtained from Sigma-Aldrich.

### Synthesis of HA-PEG-Lipid with lipid type as variable

For the synthesis of various HA-PEG-Lipid conjugates, we used three different types of Lipid-PEG-NH_2_ candidates: DMPE-PEG-NH_2_, DSPE-PEG-NH_2_, and Chol-PEG-NH_2_. Each Lipid-PEG-NH_2_ was conjugated to HA backbone by an EDC/NHS coupling reaction. Briefly, 100 mg of HA (Mw 60 k, 10 mg/mL, 1 equivalent) and an excess amount of EDC and NHS (0.075 mmol, respectively) were dissolved in 8 mL of distilled water (DW). The resulting solution was stirred at room temperature (RT) for 6 h. Subsequently, 0.05 mmol of Lipid-PEG-NH_2_ solubilized in 2 mL DMF was added with a catalytic amount of DMAP. The mixture was stirred at RT for 48 h. Afterward, the reactant was dialyzed (MWCO 12–14 kDa) in DW for 3 days to remove impurities. After lyophilization, HA-PEG-Lipid conjugates were obtained in a powder form with a yield of 70–80%. The synthesized HA-PEG-Lipid was confirmed by FTIR (FTIR, Perkin Elmer FTIR Spectrum Two, PerkinElmer, USA) and ^1^H-NMR (500 MHz FT-NMR spectrometer, Bruker, Germany) analyses.

### Fluorescence labeling of HA-PEG-Lipid

To evaluate hydrophobicity of each conjugate and characterize HALipid-NK cells, Alexa Fluor 488 hydrazide dye (Invitrogen) was additionally conjugated to each HA-PEG-Lipid. Here, the Alexa Fluor 488 hydrazide dye was conjugated with the remaining carboxylic acid group of HA-PEG-Lipid. After 1 mM of Alexa Fluor 488 hydrazide solution was slowly added to HA-PEG-DMPE, HA-PEG-DSPE, or HA-PEG-Chol pre-activated with EDC, the reaction mixture was stirred at RT for 24 h in a dark condition. After that, reactants were dialyzed (MWCO 12–14 kDa) in DW for 24 h under a dark condition and lyophilized. The dye-conjugated HA-PEG-Lipid (Fluor-HA-PEG-Lipid) was stored at − 80 °C before use. Additionally, the fluorescence was conjugated to HA (Fluor-HA) in the same method as above.

### Partition coefficient measurement of HA-PEG-Lipid

The partition coefficient (log P) of each synthesized HA-PEG-Lipid was measured in a 1:1 (v/v) mixture of octanol and aqueous 4-(2-Hydroxyethyl)piperazine-1-ethanesulfonic acid (HEPES) buffer (10 mM, pH 7.4) [23, 24]. First, Fluor-HA-PEG-Lipid and Fluor-HA were dissolved in HEPES buffer (1 mg/mL) and mixed with 1 mL of octanol (Sigma-Aldrich). After thorough vortexing for 30 min in a light-free environment, the mixture underwent overnight agitation on a shaker table, followed by a 3 h resting period at 4 °C. The octanol layer and HEPES buffer layer were carefully separated by centrifugation. Fluorescence intensity of the dye-conjugated material was then measured using microplate spectrophotometry (Ex/Em = 485/535 nm wavelength). For quantification of hydrophobic/hydrophilic balance of each lipid conjugate, log P values were calculated using the following formula:

$$Log\;P\;=\;log \frac{Fluorescence\;intensity\;of\;dye\;in\;octanol\;layer}{Fluorescence\;intensity\;of\;dye\;in\;HEPES\;buffer\;layer}$$

### Cell culture

NK-92mi cells (American Type Culture Collection, ATCC, USA) were cultured in Minimum Essential Medium Alpha (MEMα, Gibco, USA) supplemented with 12.5% horse serum (Gibco), 12.5% fetal bovine serum (FBS, Gibco), 1% penicillin–streptomycin solution (Corning, USA), 0.2 mM inositol (Sigma-Aldrich, USA), 0.1 mM β-mercaptoethanol (Sigma-Aldrich), and 0.02 mM folic acid (Sigma-Aldrich). Both MDA-MB-231 (ATCC) and human fibroblasts (hfibroblasts, Lonza, USA) were cultured in growth media consisting of 89% Dulbecco’s modified Eagle’s medium (DMEM, Corning), 10% FBS (Corning), and 1% penicillin–streptomycin solution (Corning, USA). All cells were incubated at 37 ºC with 5% CO_2_ and 95% humidity.

### Characterization of HALipid-NK cells

For characterization of HALipid-NK cells, NK cells were coated by Fluor-HA-PEG-DMPE (Fluor-HADM-NK cells), Fluor-HA-PEG-DSPE (Fluor-HADS-NK cells), and Fluor-HA-PEG-Chol (Fluor-HACH-NK cells). Briefly, NK cells (1 × 10^6^ cells) were incubated with 200 μL of each Fluor-HA-PEG-Lipid coating solution (1 mg/mL in MEMα) at RT for 30 min. After incubation, HALipid-NK cells coated with Fluor-HA-PEG-Lipid (Fluor-HALipid-NK cells) were washed twice with 1 mL of MEMα. Morphological images of Fluor-HALipid-NK cells were confirmed by fluorescence microscopy (Ti-E System, Nikon, Japan). Obtained images were analyzed by ImageJ (National Institutes of Health, USA). To determine NK cell coating efficacy, all Fluor-HALipid-NK cells were lysed by RIPA buffer (ELPIS-BIOTECH, Republic of Korea). After 30 min of incubation with RIPA buffer at 4 ºC, equal volume of DW was added to prevent foaming bubbles. The fluorescence intensity of collected supernatant was measured using microplate spectrophotometry (Ex/Em = 485/535 nm wavelength). A standard curve correlating concentration with Fluor-HA-PEG-Lipid was prepared for each group. Cell coating efficacy was subsequently calculated using this standard curve. Considering potential interference caused by cellular components, lysates of NK cells were used. Additionally, to examine the retention of cell membrane anchoring of materials, NK cells were coated with 1 mg/mL of Fluor-HA-PEG-Lipid as previously described. Mean fluorescence intensity (MFI) of Fluor-HALipid-NK cells was then measured at 0, 15, 30, 45, and 60 min using a flow cytometer (Beckman Coulter, USA).

### Assessment of NK cell reactivity by coating

To evaluate the potential for uncontrolled inflammatory responses caused by coating, NK cells were coated with HA-PEG-DMPE (HADM-NK cells), HA-PEG-DSPE (HADS-NK cells), and HA-PEG-Chol (HACH-NK cells), respectively. Additionally, NK cells were treated with 1 μg/mL of lipopolysaccharide (LPS, from *Escherichia coli* O26:B6, Sigma-Aldrich) as a positive control [14]. After 24 h of incubation, the supernatant was collected and the amount of secreted interferon‐gamma (IFN‐γ) from NK cells was quantified by ELISA (PeproTech, USA) in accordance with the manufacturer's instructions. Furthermore, the availability of native receptors/ligands on HALipid-NK cells was evaluated, focusing on representative tumor necrosis factor-related apoptosis-inducing ligand (TRAIL) and Fas ligand (FasL), both of which play essential roles in cancer recognition [25, 26]. To assess the availability of native ligands on HALipid-NK cell, NK cells or HALipid-NK cells were treated with APC-conjugated TRAIL and FasL antibodies (BD Bioscience, USA), respectively [14]. After incubation at 4 °C for 1 h, cells were washed three times with cold Dulbecco’s phosphate-buffered saline (Corning). Fluorescence signals of NK cells treated with antibodies were detected by flow cytometry.

### Viability of HALipid-NK cells

To assess the viablity of HALipid-NK cells, NK cells were coated with HA-PEG-Lipid solutions at a concentration of 1 mg/mL. NK cells or HALipid-NK cells were seeded into 96-well plates at a density of 1 × 10^5^ cells/well. NK cell viability was determined at 0, 24, and 48 h by CellTiter-Blue assay (Promega, USA), according to the manufacturer’s protocol. The final fluorescence intensity was measured using microplate spectrophotometry (Ex/Em = 560/590 nm wavelength).

### Augmented recognition ability of HALipid-NK cells toward target cancer cells

To assess the CD44 specific effector/target cluster formation, CD44-overexpressing cancer cells (MDA-MB-231) or CD44 low expressing normal cells (hfibroblasts) were co-incubated with effector NK cells. Prior to co-culture, NK cells were pre-stained with 0.1 μM Calcein-AM (Thermo Fisher Scientific) and then coated with a 1 mg/mL of HA-PEG-Lipid solution, as previously described. Simultaneously, 5 × 10^5^ of MDA-MB-231 cells or hfibroblasts were pre-stained with 1 μM of CellTracker™ Red CMTPX Dye (Invitrogen). Effector cells (5 × 10^5^ cells) were co-cultured with an equal number of target cells (5 × 10^5^ cells) (i.e., 1:1 of E:T ratio), and subsequent cluster formation between effector cells and target cells was quantified using flow cytometry. The regions displaying double positivity, with both green-fluorescent NK cells and red-fluorescent target cells, were considered indicative of cluster formation.

### In vitro cancer-killing efficacy

Cancer-killing efficacy of HALipid-NK cells was measured using a Calcein release assay. First, 1 × 10^4^ target cells (MDA-MB-231 and hfibroblasts) were stained with 15 µM of Calcein-AM solution at 37 °C for 30 min, washed twice with 1 mL of MEMα and then co-cultured at 37 °C with NK cells or HALipid-NK cells at a 10:1 effector to target (E:T) ratio (i.e., 100,000 NK cells and 10,000 target cells) for 4 h. After incubation, fluorescence intensity of the collected supernatant was measured using microplate spectrophotometry (Ex/Em = 485/535 nm wavelength). The spontaneous release value represents the intensity of Calcein released from target cells without co-culture, while the maximum lysis value was obtained after treatment with 5% Triton X-100 (Sigma Aldrich) to ensure complete cell lysis under controlled conditions. The percent of specific cell lysis was calculated with the following formula:

$$\begin{aligned} Specific\;cell\;lysis\;\left( \% \right)\, = & \,\left( {Experiment\;release\; - \;Spontaneous \, release} \right) \, \\ / & \left( {Maximum \, release\; - \;Spontaneous \, release} \right)\, \times \,100 \\ \end{aligned}$$

### Statistical analysis

All quantitative data are presented as mean ± standard deviation. All statistical analyses and graph plotting were conducted using GraphPad Prism V 7.0 (GraphPad Software Inc., USA). To consider significant differences, all in vitro quantitative data were analyzed by one-way analysis of variance (ANOVA) with Tukey’s post-hoc test. Statistical differences were considered when p-values were less than 0.05.

## Results and discussion

### Synthesis and characterization of HA-PEG-Lipid with different lipid type

**Fig. 1:**
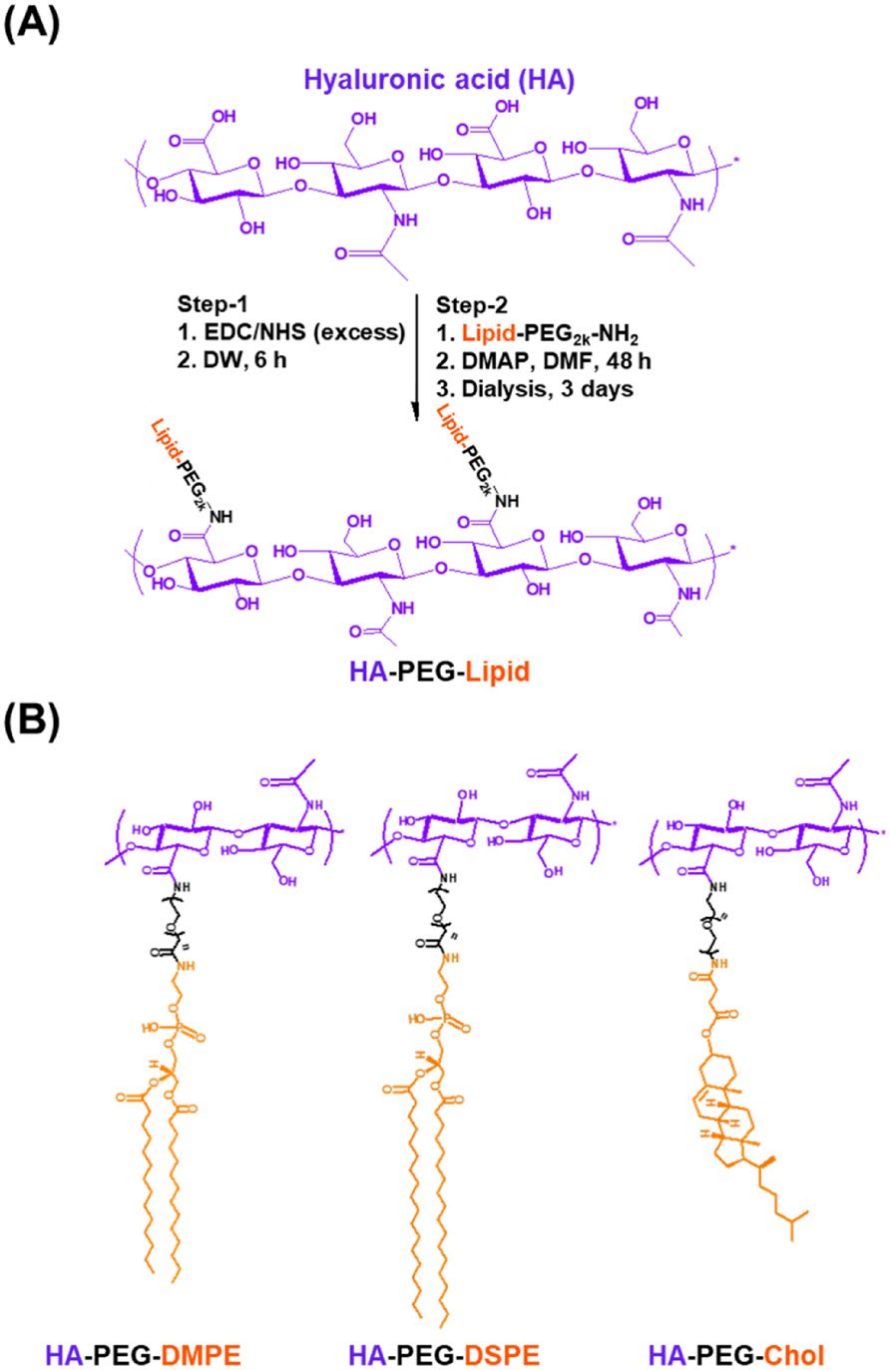
Synthesis procedure of HA-PEG-Lipid. **A** Conjugation of Lipid-PEG-NH_2_ to the carboxylic acid in the HA backbone using EDC/NHS chemistry. **B** Chemical structures of HA-PEG-Lipid with three different lipid types (i.e., HA-PEG-DMPE, HA-PEG-DSPE and HA-PEG-Chol)

In this study, we hypothesized that NK cells could effectively recognize and eliminate CD44-overexpressing cancer cells when adequately coated with HA-conjugated with appropriate lipid candidates. Generally, the capacity of a lipid anchor to effectively tether to the cell membrane depends on (1) the hydrophobicity of the lipid and (2) the structural resemblance to the lipid components of the cell membrane. Specifically, hydrophobicity is governed by the length of the carbon chain and the incorporation of tail-pulling lipids. Furthermore, given that two-tailed lipids are predominantly found in cell membranes, the utilization of such lipids (i.e., two tail lipid) would confer advantages for cell surface anchoring [10]. Vitamin E has been considered as a potential candidate for cell surface anchoring. In a study conducted by Yang et al., various hydrophobic materials were tested for their ability to insert into NK cell surfaces. Interestingly, both the single alkyl chain (C18) and vitamin E were unable to immobilize on the NK cell surface [27]. Moreover, Liu et al., compared the anchoring efficacy on B16F10 cells by adjusting the lipid module of lipid-oligonucleotides with (1) C18 one tail lipid, (2) C18 two tail lipid, and (3) Chol [22]. Consequently, the C18 two tail material with robust hydrophobicity and similarity to cell membrane lipids, exhibited the highest anchoring efficacy for B16F10 cells compared to C18 one tail lipid and Chol. However, given the complex and dynamic nature of cell membranes, it is essential to identify appropriate lipid candidates for each specific cell type. For instance, DMPE (C14) exhibits higher surface anchoring efficacy on hepatocytes than 1,2-dipalmitoyl-sn-glycerol-3-phosphatidylethanolamine (C16) and DSPE (C18), despite having lower hydrophobicity [28]. Moreover, Chol shows homogeneous surface anchoring on RAW 264.7 cells, while DSPE does not anchor on RAW 264.7 cells [29]. Hence, it is imperative to meticulously choose appropriate lipids for tailoring surface engineering of NK cells. Herein, we selected DMPE, DSPE, and Chol commonly used for cell surface engineering. We then conjugated HA to these lipid candidates to bestow CD44 targeting functionality to NK cells (Fig. 1A). Specifically, Lipid-PEG-NH_2_ was conjugated to the carboxylic acid in the HA backbone through an EDC/NHS coupling reaction. The degree of lipid substitution was approximately 18‒19% based on ^1^H-NMR data (Fig. 1B).

Chemical structures of synthesized HA-PEG-DMPE, HA-PEG-DSPE and HA-PEG-Chol were analyzed by FTIR and ^1^H-NMR. FTIR analysis of various HA-PEG-Lipid materials demonstrated effective linkage of Lipid-PEG-NH_2_ to carboxylic moieties of HA backbone. FTIR spectra and a detailed comparative analysis of IR vibrational frequencies are shown in Additional file 1: Figs. S1–S3. New IR signals (corresponding to newly formed amides) were observed between 1647 and 1649 cm^–1^ in HA-PEG-DMPE, HA-PEG-DSPE and HA-PEG-Chol, indicating successful conjugation of Lipid-PEG-NH_2_ with the HA backbone. On the other hand, representative IR frequencies of Lipid-PEG-NH_2_ at 2883 cm^–1^ (CH stretching for Chol), 2884 and 2925 cm^–1^ (CH stretching for DMPE), and 2850, 2884, and 2916 cm^–1^ (CH stretching for DSPE) were also detected with HA peaks in IR spectra of HA-PEG-Lipid (Additional file 1: Figs. S1–S3). After comparative analysis of each spectrum, HA-PEG-Lipid clearly showed IR peaks of both Lipid-PEG-NH_2_ and HA, confirming that Lipid-PEG-NH_2_ was successfully linked to HA.

**Fig. 2:**
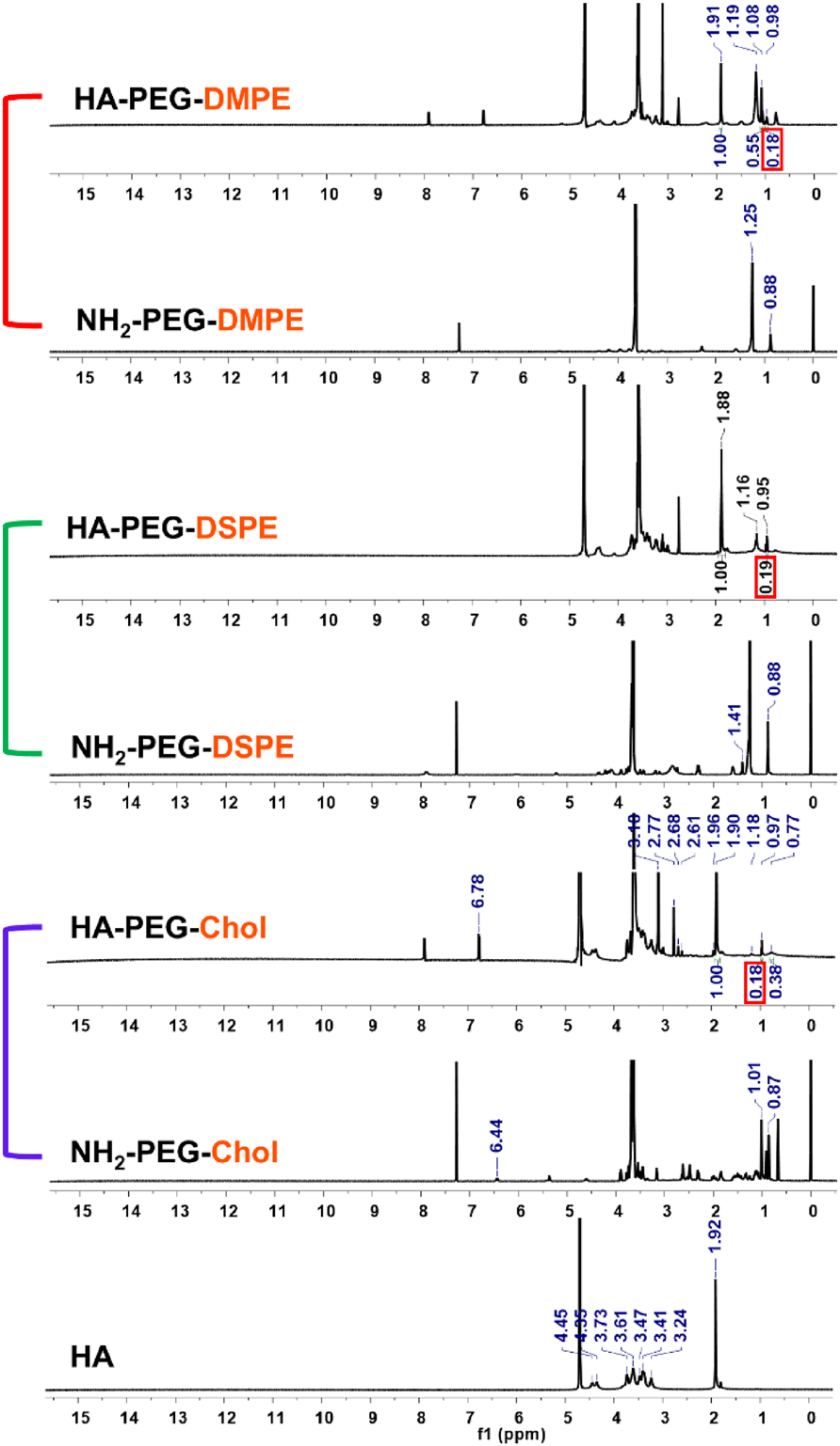
Proton nuclear magnetic resonance (^1^H-NMR, 500 MHz) spectra. D_2_O was used as NMR solvent for HA, HA-PEG-DMPE, HA-PEG-DSPE, and HA-PEG-Chol. CDCl_3_ was used as NMR solvent for DMPE-PEG-NH_2_, DSPE-PEG-NH_2_, and Chol-PEG-NH_2_. The degree of Lipid-PEG-NH_2_ conjugation in HA backbone was calculated by peak integration between the N-acetyl group of HA (δ1.9 ppm) versus the terminal –CH_3_ of lipids (δ0.8–0.95 ppm). Red box indicates the degree of lipid substitution

Afterwards, the addition of Lipid-PEG-NH_2_ in HA backbone and the formation of final HA-PEG-Lipid biomaterials were further confirmed by ^1^H-NMR analysis. According to Fig. 2, HA presents the following NMR signals: (500 MHz, D_2_O) N-acetyl COCH_3_ (δ1.92 ppm), CH_2_‒ in HA repeating units (δ3.24‒3.73 ppm), primary hydroxyl moiety (δ4.35 ppm), and secondary hydroxyl moiety attached with a ring (δ4.45 ppm). The introduction of Lipid-PEG-NH_2_ to HA was confirmed by the presence of proton signals from Lipid-PEG-NH_2_ and HA. NMR signals of HA-PEG-DMPE were observed as follows: (500 MHz, D_2_O) ‒CH_3_ of terminal alkyl chain (δ0.98 ppm), ‒CH_2_ groups of the alkyl chain of DMPE (δ1.08–1.19 ppm), *N*-acetyl COCH_3_ group attached with glucosamine rings of HA (δ1.91 ppm), CH_2_ near to ester moiety of DSPE (δ2.78 ppm), CH_2_ group between phosphate and DMPE-PEG-NH_2_ (δ3.11 ppm), CH_2_ protons of glucuronic and glucosamine rings belonging to HA (δ3.25–3.50, and δ3.68–3.79 ppm), ethyl oxide units of PEG (δ3.61 ppm), and primary and secondary OH groups of glucose rings of HA (δ4.10–4.44 ppm). HA-PEG-DSPE showed proton NMR signals as follows: (500 MHz, D_2_O) terminal ‒CH_3_ groups of DSPE alkyl chain (δ0.95 ppm), ‒CH_2_ groups of DSPE alkane chain (δ1.14–1.17 ppm), *N*-acetyl COCH_3_ moiety of HA glucosamine rings (δ1.88 ppm), CH_2_ near ester, phosphate, and amide in lipid (δ2.96–3.10 ppm), CH_2_ protons of glucuronic and glucosamine rings in HA (δ3.23–3.49, and δ3.65–3.72 ppm), PEG units belonging to DSPE-PEG-NH_2_ (δ3.58 ppm), and primary and secondary OH groups of glucose rings of HA (δ4.08–4.43 ppm). Similarly, HA-PEG-Chol showed NMR signals as follows: (500 MHz, D_2_O) ‒CH_3_ belonging to Chol alkyl chain (δ0.97 ppm), ‒CH_2_ and ‒CH groups of Chol alkyl chain (δ1.18–1.79 ppm), N-acetyl COCH_3_ group of HA (δ1.90 ppm), protons of CH_2_ groups to between PEG and Chol (δ2.61–2.77 ppm), PEG OCH_2_CH_2_ units (δ3.60 ppm), CH_2_ protons of glucuronic and glucosamine rings belonging to HA (δ3.23–3.49, and δ3.67–3.73 ppm), and primary and secondary hydroxyl groups protons (δ4.37 and 4.45 ppm). The degree of lipid substitution (i.e., conjugated Lipid-PEG-NH_2_ moieties per 100 sugar residues in HA backbone) was calculated using NMR spectra analysis based on signals at δ1.9 ppm (N-acetyl COCH_3_ moiety) and δ0.9 ppm (CH_3_ groups of DMPE, DSPE, and Chol) (Fig. 2).

Hydrophobicity of synthesized HA-PEG-Lipid conjugates (i.e., HA-PEG-DMPE, HA-PEG-DSPE, and HA-PEG-Chol) was determined by measuring their log P values in comparison to HA following a previously reported method [23]. The log P value of HA was − 3.31 ± 0.07. After conjugation of Lipid-PEG-NH_2_ with HA backbones, log P value was − 2.80 ± 0.07 for HA-PEG-DMPE, − 1.96 ± 0.07 for HA-PEG-DSPE, and − 2.40 ± 0.35 for HA-PEG-Chol (Additional file 1: Figs. S4). In contrast to HA, which displayed the lowest log P values due to its high hydrophilicity, these increased log P values of different lipid-binding biomaterials indicated successful conjugation of lipids into the HA backbone for anchoring HA to NK cell surface.

### Characterization of HALipid-NK cells

**Fig. 3:**
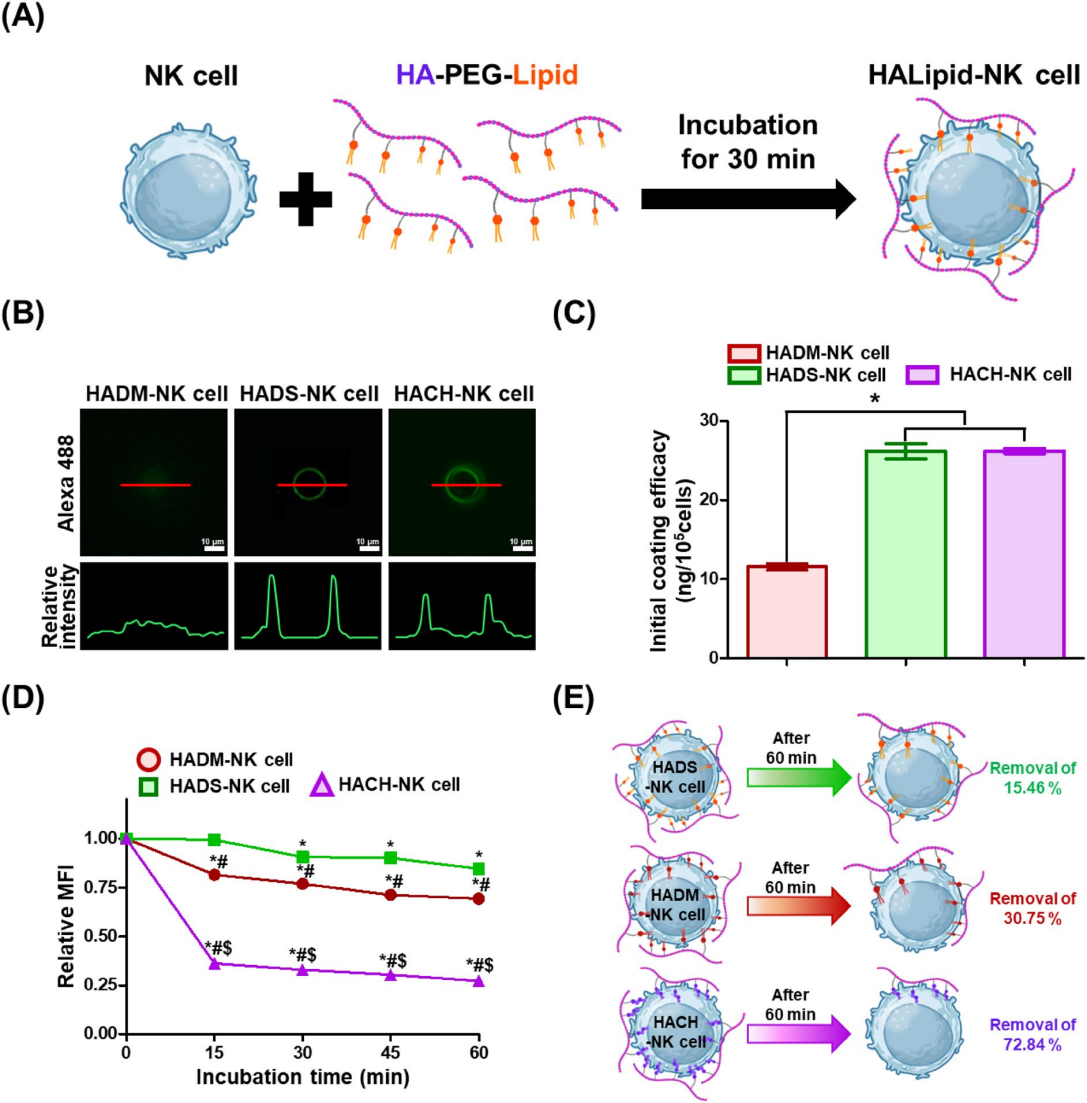
Characterization of HALipid-NK cells. **A** Schematic representation of NK cell coating with HA-PEG-Lipid and fabricated HALipid-NK cells. First, 1 × 10^6^ NK cells were coated with 200 μL of Fluor-HA-PEG-Lipid coating solution (1 mg/mL) at RT for 30 min. After washing, Fluor-HALipid-NK cells were visualized via fluorescence microscope. **B** Fluorescence images of HADM-NK cell, HADS-NK cell, and HACH-NK cell. Obtained images were analyzed with ImageJ. **C** Quantitative assessment of NK cell coating performance of HALipid-NK cells. (*) indicates a significant difference compared to HADM-NK cells (*p* < 0.05). **D** Retention of cell membrane anchoring of Fluor-HA-PEG-Lipid detected by flow cytometry at 0, 15, 30, 45, and 60 min. The relative mean fluorescence intensity (MFI) was assessed based on the MFI value at 0 min. (*) indicates a significant difference compared to 0 h for each group (*p* < 0.05). (#) indicates a significant difference compared to HADS-NK cells at the same time point (*p* < 0.05). ($) indicates a significant difference compared to HADM-NK cells at the same time point (*p* < 0.05). **E** Illustration depicting the average amount of HA-PEG-Lipid removal from NK cells after 60 min

Through simultaneous hydrophobic interaction between lipid conjugates and NK cell membranes, NK cells could be easily coated after incubation with HA-PEG-Lipid at RT for 30 min (Fig. 3A). NK cell membranes were homogeneously coated with Fluor-HA-PEG-Lipid when DSPE and Chol were used as lipid anchor moieties, while cytoplasmic internalization of Fluor-HA-PEG-DMPE was observed (Fig. 3B). Additionally, a quantitative assessment of NK cell coating performance revealed that both Fluor-HADS-NK cells (26.18 ng/10^5^ cells) and Fluor-HACH-NK cells (26.21 ng/10^5^ cells) exhibited higher NK cell coating efficacies than Fluor-HADM-NK cells (11.55 ng/10^5^ cells) (Fig. 3C). It was assumed that this variation in coating efficacy was influenced by the hydrophobicity of materials used, with DSPE and Chol-based materials demonstrating higher hydrophobicity than DMPE (Additional file 1: Fig. S4).

For successful cancer treatment, it is imperative that materials anchored to the cell membrane remain stable on the NK cell surface as NK cells will circulate in the bloodstream following intravenous injection, reach the tumor site, recognize the target, and initiate the attack. Thus, fluorescence intensity of Fluor-HALipid-NK cells was tracked using flow cytometry at 15 min intervals for 60 min (Fig. 3D). During the initial 15 min, HA-PEG-Chol was swiftly removed from the NK cell membrane, resulting in a removal rate of 63.80% for coating materials, despite the initially higher coating efficacy.

In contrast, HA-PEG-DSPE exhibited longer surface maintenance after the initial coating process compared to HA-PEG-DMPE and HA-PEG-Chol. Specifically, 30.75% of HA-PEG-DMPE and 72.84% of HA-PEG-Chol were removed from the NK cell surface within 60 min (Fig. 3E). In contrast, only 15.46% of HA-PEG-DSPE was discarded from the NK cells surface after 60 min of incubation. The long-term retention ability of DSPE compared to DMPE is closely related to their hydrophobicity. Hydrophobic biomaterials anchor to the cell membrane via hydrophobic interactions. Consequently, HA-PEG-DSPE with longer lipid carbon chain (i.e., higher hydrophobicity) exhibited prolonged retention on the NK cell surface compared to HA-PEG-DMPE. Moreover, Chol could be rapidly exchanged into NK cell cytoplasm by the Chol delivery pathway [27, 30]. Mammalian cells typically acquire Chol through the receptor-mediated endocytosis of low-density lipoproteins (LDLs), which predominantly consist of Chol fatty acid esters. Once lysosomal acid lipase hydrolyzes cholesteryl fatty acid esters into free Chol, it activates the endocytic Chol transport routes, facilitating Chol uptake via receptors present on the cell surface [31, 32]. Similar results were also exhibited in the previous study [27]. In this study, Yang et al., synthesized aptamer-lipid (two-tail C18 hydrocarbon chains or Chol) conjugates for the NK cell surface engineering. As a result, aptamer-Chol rapidly disappeared from the NK cell surface within 15 min (> 80% reduction). On the other hand, the two-tail C18 hydrocarbon chains stably remained (> 90% remained) on the NK cell surface for 60 min. Hence, DSPE mediated biomaterials with robust hydrophobicity and enduring retention capability emerge as dependable lipid candidates for engineering NK cell surfaces. Furthermore, this notable anchoring attribute holds significance in upholding the steadfast presence of NK cells on surfaces, particularly under dynamic blood flow conditions upon their introduction into the body. Therefore, HADS-NK cells could be utilized as a promising and effective cell therapy agent that reliably reaches tumors post-administration into the body, recognizes specific targets, and effectively eliminates them.

### Preservation of surface signaling components in HALipid-NK cells

**Fig. 4:**
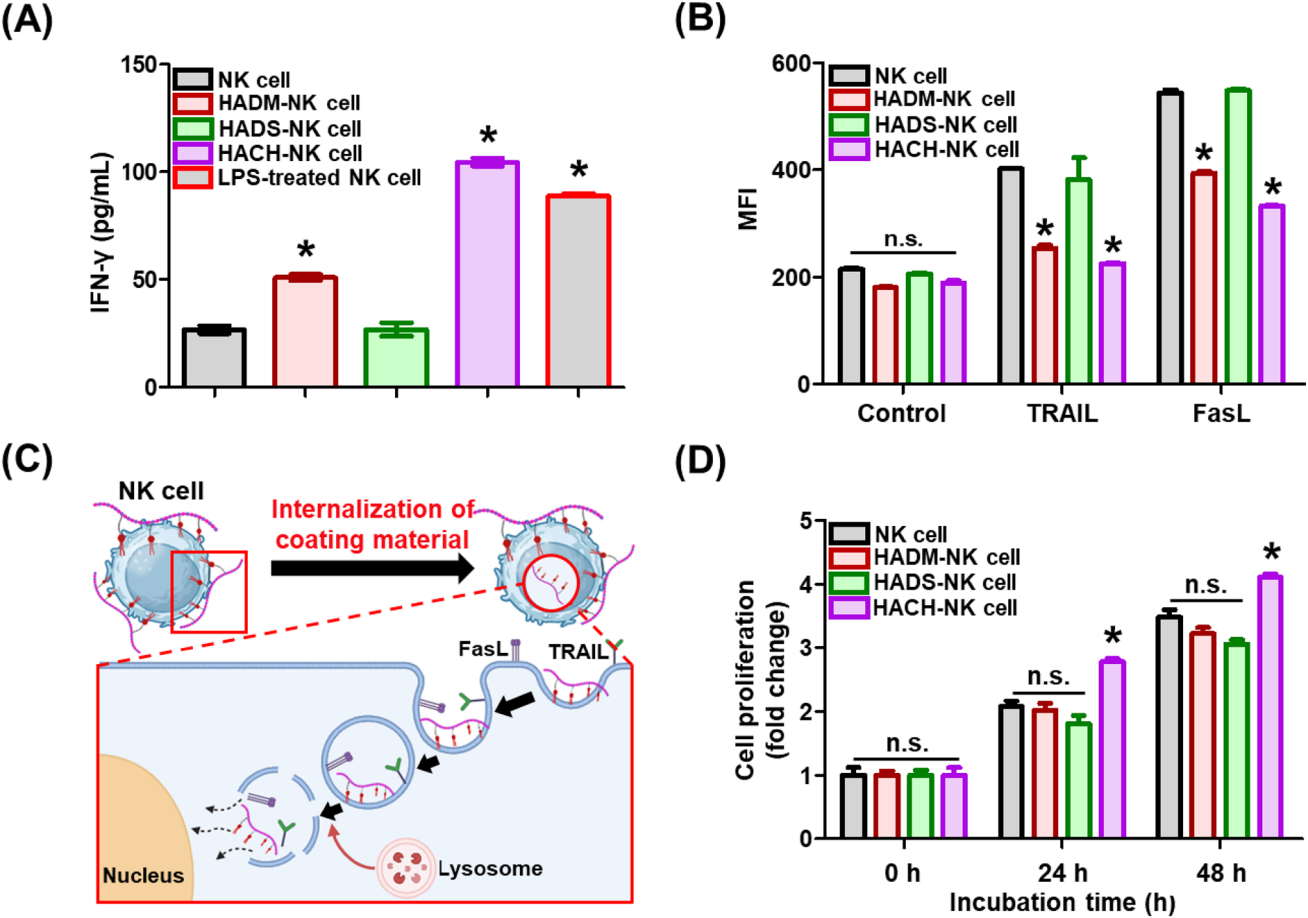
Reactivity of NK cells following surface coating. **A** Quantification of IFN-γ secretion from NK Cells and HALipid-NK cells. A positive control group was established, wherein NK cells were exposed to lipopolysaccharide (LPS) to stimulate an inflammatory response associated with IFN-γ secretion. The secreted IFN-γ was quantified by ELISA. (*) indicates a significant difference compared to NK cells and HADS-NK cells (*p* < 0.05). **B** Availability of native ligands on HALipid-NK cells. Tumor necrosis factor-related apoptosis-inducing ligand (TRAIL) and Fas ligand (FasL) were selected as essential ligands on NK cells for cancer recognition. NK cells were treated with APC-conjugated TRAIL and FasL antibodies immediately after coating. Fluorescence signals of antibodies were detected using flow cytometry. (*) indicates a significant difference compared to NK cells and HADS-NK cells (*p* < 0.05) (**C**) Schematic illustration of the internalization of coating materials (specifically, both HA-PEG-DMPE and HA-PEG-Chol) anchored on the NK cell surface. **D** Proliferation of NK cells coated with a 1 mg/mL of HA-PEG-Lipid solution. Proliferation levels were measured at 0, 24, and 48 h by CellTiter-Blue assay. (*) indicates a significant difference compared to NK cells, HADS-NK cells, and HADM-NK cells (*p* < 0.05) at the same time point. (n.s.) indicates no statistically significant difference between experimental groups

To achieve successful and safe anticancer treatment, it is imperative to precisely control the immune response while preserving innate properties of NK cells. In this study, we conducted an evaluation to ascertain whether application of coating materials and the coating process to NK cells induced unintended inflammatory responses. Among various cytokines secreted by NK cells, IFN-γ is a representative cytokine produced by activated NK cells. It plays a pivotal role in preventing early infections and promoting adaptive immunity [33, 34]. Nonetheless, uncontrolled sporadic release of IFN-γ could also lead to inflammation and potentially contribute to tumor metastasis [35, 36]. Therefore, levels of IFN-γ in HALipid-NK cells were quantified and compared to those in NK cells (Fig. 4A). LPS is an outer membrane component of Gram-negative bacteria and serves as a representative pathogen-associated molecular pattern (PAMP) factor [15, 37]. LPS directly influences NK cells by engaging Toll-like receptor 4 (TLR4) on the NK cell surface. The initial signaling pathway is initiated at the plasma membrane upon the interaction of TLR4 with LPS [38]. Subsequently, the TLR4-LPS complex is internalized into the cell membrane, leading to the activation of downstream signaling pathways. The secretion of IFN-γ from the cell is promoted through a series of downstream cell signaling pathways. Hence, the group of NK cells treated with LPS was employed as a positive control.

Results showed that HADS-NK cells secreted similar amounts of IFN-γ to control NK cells, while coating with HA-PEG-DMPE or HA-PEG-Chol resulted in higher IFN-γ production, similar to the positive control group (i.e., LPS-treated NK cells). Conversely, NK cells coated with HA-PEG-DSPE exhibited a comparable level of IFN-γ secretion as regular NK cells. This result suggests that HA-PEG-DSPE does not induce an unintended inflammatory response in NK cells.

Next, the availability of surface ligands (TRAIL and FasL) of HALipid-NK cells was evaluated based on binding with APC-labeled antibodies and quantification using flow cytometry (Fig. 4B). HADS-NK cells exhibited similar MFI levels to non-treated NK cells, demonstrating no disturbance in antibody-NK cell membrane ligand interaction. Therefore, HADS-NK cells did not structurally interfere with signal binding cascades during the coating process. However, HADM-NK cells and HACH-NK cells showed lower MFI levels than NK cells, indicating architectural hindrance in membrane signaling phenomenon or substantial damages to NK cells by unexpected cytoplasmic internalization. As depicted in Fig. 3A, HA-PEG-Lipid was able to permeate into the cytoplasm of NK cells within the initial 30 min of the coating process. Furthermore, a rapid decrease of HA-PEG-Chol from 45 min after coating shown in Fig. 3D indicated that the disappearance of this Chol-conjugated coating material might be closely related to cytoplasmic penetration rather than physical dissociation. In accordance with LPS-dependent IFN-γ secretion shown in Fig. 4A, internalized coating materials (i.e., both HA-PEG-DMPE and HA-PEG-Chol) could induce downstream inflammatory response and insufficient signal binding via damages or deactivation of NK cells.

Furthermore, when HA-PEG-DMPE or HA-PEG-Chol was endocytosed into NK cells, membrane-located ligands and protein complexes could be internalized together (Fig. 4C). As a result, fewer number of ligands were available on NK cell surface as compared to non-treated NK cells or HADS-NK cells, which could further reduce binding events with signaling molecules (such as LPS) in surrounding environments.

Finally, the viability and proliferation capacity of HALipid-NK cells were verified (Fig. 4D). No cytotoxic effects were detected in experimental groups over a 48 h period. However, HA-PEG-Chol enhanced NK cell viability after 24 and 48 h of incubation compared to the control group. This observation leads to the speculation that the increased proliferation observed in HACH-NK cells might be associated with Chol-dependent facilitation of melastatin-like transient receptor potential 7 channels, initiating Ca^2+^ entry and subsequent cell proliferation [39, 40]. This finding further suggested that HA-PEG-Chol anchored on the NK cell membrane was internalized, which triggered uncontrolled reactions in NK cells. In conclusion, both HA-PEG-DMPE and HA-PEG-Chol were internalized into cell body, leading to the induction of an inflammatory response and a reduced presence of ligands. Consequently, HA-PEG-DSPE does not interfere with intrinsic functions of NK cells. Thus, it holds potential for the development of customized surface-engineered NK cell therapeutics.

### Sequential anticancer mechanism of membrane-coated NK cells

A sequential anticancer mechanism of NK cells primarily depends on the initial membrane contact with a target tumor and subsequent immunological synapse formation. Such cellular surface-mediated interaction with target cancer cells leads to further activation of NK cells, thereby facilitating the secretion of lytic granules including perforin and granzyme. These secreted granules can then induce the disruption and elimination of cancer cells. Therefore, the initial membrane-dependent interaction between NK cells and target malignant cells can regulate proper and sufficient recognition of cancer cells.

**Fig. 5:**
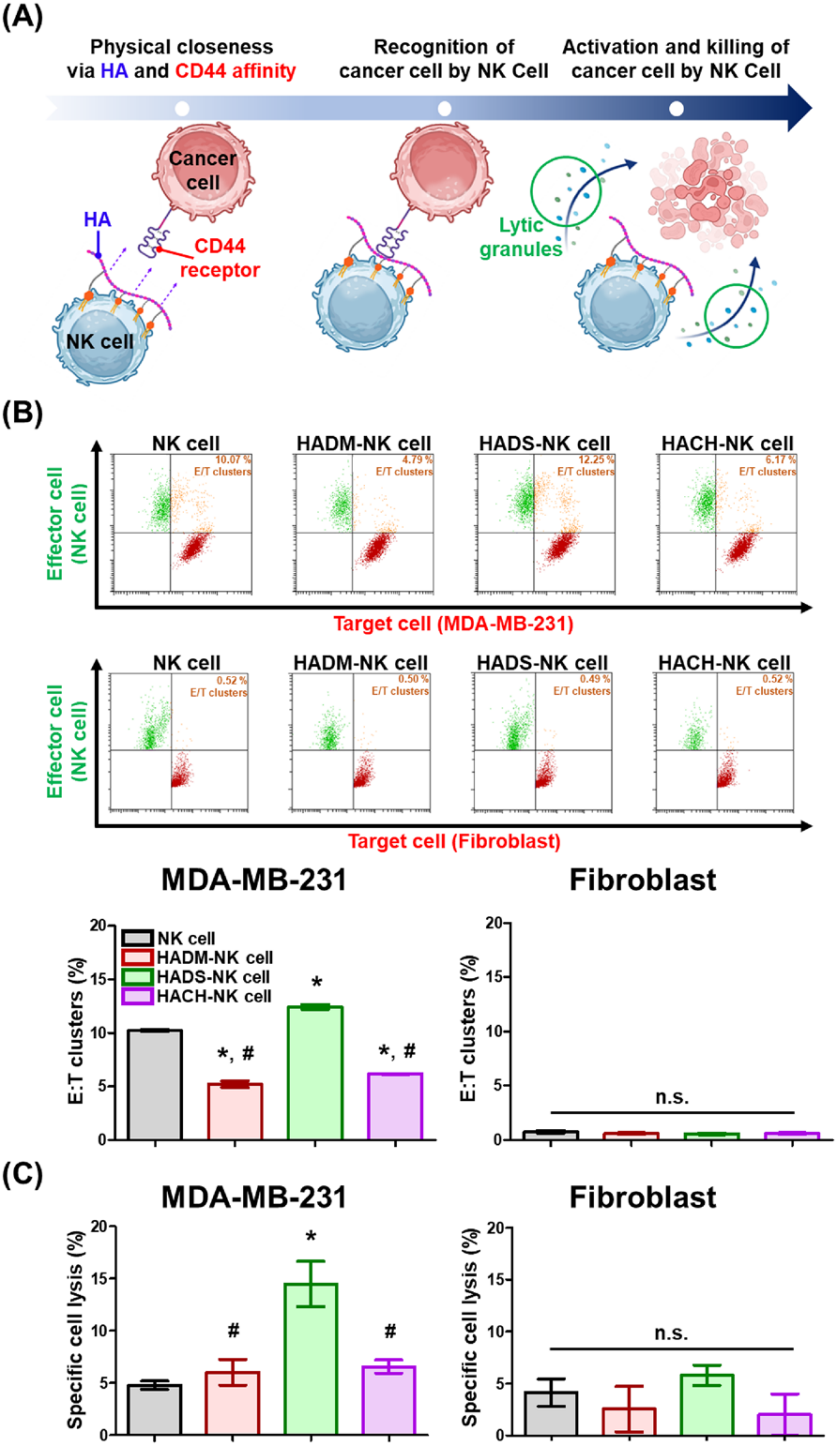
In vitro anticancer activity using HALipid-NK cells. **A** Illustration of the anticancer process involving HALipid-NK cells. HALipid-NK cells achieved physical closeness via HA and CD44 affinity, subsequently enhancing cancer recognition and initiating cancer-killing. **B** Cluster formation of effector NK cells and target cells (E/T cluster). CD44-overexpressing cancer cells (MDA-MB-231) and CD44-negative normal cells (hfibroblasts) were utilized as target cells to compare CD44-specific target recognition of HALipid-NK cells. NK cells were labeled with Calcein-AM (green). Target cells were labeled with CellTracker™ Red. Effector NK cells and target cells were co-cultured at 37 ºC for 30 min. E/T cluster formation was quantitatively analyzed as a population of double-positive cells in flow cytometry measurements. (*) indicates a significant difference compared to NK cell group (*p* < 0.05). (#) indicates a significant difference compared to HADS-NK cell group (*p* < 0.05). **C** Percentage of specific cell lysis by HALipid-NK cells. NK cells or HALipid-NK cells were co-cultured with target cells at a 10:1 effector to target (E:T) ratio at 37 ºC for 4 h. Target cell lysis was measured by a Calcein release assay. (*) indicates a significant difference compared to NK cell group (*p* < 0.05). (#) indicates a significant difference compared to HADS-NK cell group (*p* < 0.05). (n.s.) indicates no statistically significant difference between experimental groups

Based on these sequential anticancer processes, we incorporated HA-PEG-Lipid conjugate biomaterials onto NK cell surfaces for augmented cancer recognition by providing additional HA ligands for enhanced interaction with CD44 on target cancer cells (Fig. 5A). CD44, a type of transmembrane glycoprotein receptor, is upregulated in hypoxic microenvironments and overexpressed in aggressive cancer, making it an important target for killing solid tumor [41, 42]. HA-CD44 binding occurs diverse routes including (1) hydrogen bonding between the C6-OH of HA with Tyr109 of CD44, (2) hydrogen bonding between the carboxylate group in HA Tyr46 and Arg45 of CD44, (3) hydrogen bonding between the vicinal diols of HA and the residues (Arg45, Arg82) in CD44, (4) hydrophobic interaction between the N-acetyl group in HA with Tyr83, Ile92, and (5) disulfide bond between Cys81 and Cys101 of CD44 [43–45]. Leveraging this array of specific binding capabilities, numerous earlier studies have developed drug delivery systems utilizing HA to target CD44 on the surface of solid tumors [46]. Therefore, the well-established CD44-targeting HA, protruding from the NK cell membrane, exhibited specific binding to CD44 markers highly prevalent on the cancer cell surface, thereby instigating a robust affinity between NK cells and cancer cells [47]. Hence, CD44-postive cancer cell (i.e., MDA-MB-231) was selected as an in vitro target cancer model to investigate HA-CD44 dependent cancer recognition of our HALipid-NK cells [48, 49]. Optimized lipid anchor moieties conjugated in HA-PEG-Lipid were further evaluated in terms of augmented immunological synapse formation (i.e., target recognition via membrane contact) and cancer killing efficacy (i.e., tumor elimination).

To evaluate the target cancer specific recognition ability of HALipid-NK cells specifically via improved physical interaction by lipid conjugates, NK cells were co-cultured with target cells (MDA-MB-231 and hfibroblasts) at a 1:1 of E:T ratio. Cell mixtures were then analyzed by flow cytometry (Fig. 5B). Each cell line was distinguished through individual fluorescence staining. NK cells were labeled with Calcein AM (green). Target cells were labeled with CellTracker™ Red (red). As a result, HADS-NK cells exhibited the highest level of cluster formation (12.25%) as compared to other lipid anchor groups when co-cultured with MDA-MB-231. Additionally, within all experimental groups, NK cells did not display cluster formation when cultured with normal hfibroblasts (approximately 0.5% of clusters). This observation could be attributed to ability of NK cell to differentiate between normal and cancer cells, with HA presented on the surface of NK cells through coating without inducing unwanted off-target interactions with normal cells.

Moreover, cancer killing activity of HALipid-NK cells was evaluated by Calcein release assay (Fig. 5C). At a 10:1 of E:T ratio, HADS-NK cells exhibited significantly enhanced specific cancer cell lysis (17.38%) toward CD44-positive MDA-MB-231 cancer cell. In addition, off-target killing effect against hfibroblasts was not observed in any NK cell groups. In accordance with E/T cluster formation (i.e., target recognition ability) in Fig. 5B, these results also demonstrated that (1) a sequential anticancer mechanism of our HALipid-NK cells was achieved by the surface incorporation of HA-PEG-DSPE rather than by other types of anchors and that (2) selective immunological synapse formation was obtained by surface-decorated HA onto NK cells and CD44 on cancer cells.

## Conclusions

In this study, we developed HALipid-NK cells with augmented anticancer efficacies through optimization of membrane-inserting lipid anchor in HA-PEG-Lipid conjugate biomaterials. Due to the dynamic and complex structure of cell membranes, it was crucial to establish a stable hydrophobic interaction between HA-PEG-lipid conjugates and NK cell surfaces. The HA-CD44 affinity between HALipid-NK cells and cancer cells could improve the cancer recognition ability of surface-modified NK cells and subsequently induce the immunological synapse formation, resulting in enhanced cancer killing.  In terms of high coating efficacy and durable retention onto NK cell membrane, HADS-NK cells exhibited higher anticancer efficacy than HADM-NK cells and HACH-NK cells. Our results demonstrate that a suitable modification in material design for lipid structure-dependent hydrophobic interaction is an important controlling parameter in biomaterial-mediated ex vivo NK cell surface engineering. This modular design of HA-PEG-DSPE could be utilized so that various cancer-targeting immune cell engineering techniques in adoptive cell therapy could be further developed by switching cancer recognition end unit of lipid conjugates.

### Supplementary Information

**Additional file 1: Figure S1. **FTIR spectra of HA, DMPE-PEG-NH_2_ and synthesized HA-PEG-DMPE. [3396 cm^–1^ OH stretching of HA, 3269 cm^–1^ NH stretching of newly formed amide and DMPE-PEG- amides, 2925 cm^–1^ symmetric and asymmetric CH stretching of DMPE chain, 2883 and 2848 cm^–1^ CH stretching of both HA and DMPE, 1736 cm^–1^ >C=O ester bond stretching of DMPE-PEG-NH_2_, new broad peak at 1647 cm^–1^ stretching due to newly formed amide bond, 1563–1344 cm^–1^ CH and NH bendings of both HA and DMPE, 1280 cm^–1^ C-N stretching of DMPE and newly formed amide bond, 1147, 1080 and 945 cm^–1^ C–O–C and CO stretchings of both HA and DMPE]. **Figure S2. **FTIR spectra of HA, DSPE-PEG-NH_2_ and synthesized HA-PEG-DSPE. [3398 cm^–1^ OH stretching of HA, 3274 cm^–1^ NH stretching of newly formed amide bond and DSPE-PEG- amides, 2917 cm^–1^ symmetric and asymmetric CH stretching of DSPE lipid chain, 2888 and 2851 cm^–1^ CH stretching of both HA and DSPE lipid chain, 1736 cm^–1^ >C=O ester bond stretching present in DSPE-PEG, sharp peak at 1647 cm^–1^ stretching frequency for newly formed amide bond, 1562–1344 cm^–1^ CH and NH bendings of both HA and DSPE lipid chains, 1251 cm^–1^ C-N stretching of DSPE lipid and formed amide bond between HA and DSPE lipid, 1151, 1079, 1041 and 945 cm^–1^ C–O–C and CO stretchings frequencies due to both HA and DSPE lipid]. **Figure S3. **FTIR spectra of HA, Chol-PEG-NH_2_ and synthesized HA-PEG-Chol. [3411 cm^–1^ OH stretching of HA, 3277 cm^–1^ NH stretching of amides, 2945 cm^–1^ CH stretching of chol, 2870 cm^–1^ CH stretching of both HA and chol, 1731 cm^–1^ >C=O of chol ester, broad peak at 1645 cm^–1^ due to newly formed amide bond, 1563–1302 cm^–1^ CH bendings of both HA and chol, 1250-945 cm^–1^ C–O–C and CO stretchings of both chol and HA]. **Table S1. **Detailed IR frequencies of hyaluronic acid (HA), DMPE-PEG-NH_2_, and HA-PEG-DMPE biomaterial. **Table S2**. Detailed IR frequencies of hyaluronic acid (HA), DSPE-PEG-NH_2_, and HA-PEG-DSPE biomaterial. **Table S3**. Detailed IR frequencies of hyaluronic acid (HA), Chol-PEG-NH_2_, and HA-PEG-Chol biomaterial. **Figure S4.** Log P values for HA and HA-PEG-Lipid (i.e., HA-PEG-DMPE, HA-PEG-DSPE, and HA-PEG-Chol) to assess the hydrophobicity of these amphiphilic materials.

## Data Availability

All data generated or analyzed during this study are included in this published article and its additional information files.

## References

[1] Yilmaz A, Cui H, Caligiuri MA, Yu J (2020). J. Hematol. Oncol..

[2] Kim S-H, Cho E, Kim YI, Han C, Choi BK, Kwon BS (2021). Nat. Commun..

[3] Gong Y (2022). Adv. Sci..

[4] Chiossone L, Dumas P-Y, Vienne M, Vivier E (2018). Nat. Rev. Immunol..

[5] Matosevic S (2018). J. Immunol. Res..

[6] Liu C, Lai H, Chen T (2020). ACS Nano.

[7] Melaiu O, Lucarini V, Cifaldi L, Fruci D (2020). Front. Immunol..

[8] Lee DY, Cha B-H, Jung M, Kim AS, Bull DA, Won Y-W (2018). J. Biol. Eng..

[9] Park HW, Lee CE, Kim S, Jeong W-J, Kim K (2023). Tissue Eng. Part B Rev..

[10] Wang X (2020). ACS Cent. Sci..

[11] Kim S, Kim K (2022). Biomater. Adv..

[12] Pan S, Wang F, Jiang J, Lin Z, Chen Z, Cao T, Yang L (2023). Clin. Oncol..

[13] Jangid AK, Kim S, Kim K (2023). Biomater. Res..

[14] J. Almeida‐Pinto, M. R. Lagarto, P. Lavrador, J. F. Mano, V. M. Gaspar. Adv. Sci., 2304040 (2023) 10.1002/advs.202304040 PMC10700290 37823678

[15] Jangid AK, Kim S, Kim HJ, Kim K (2023). Bioconjug. Chem..

[16] Ribeiro RD, Pal D, Jamieson D, Rankin KS, Benning M, Dalgarno KW, Ferreira AM, Appl ACS (2017). Mater. Interfaces.

[17] Kozlovskaya V, Harbaugh S, Drachuk I, Shchepelina O, Kelley-Loughnane N, Stone M, Tsukruk VV (2011). Soft Matter.

[18] Lingwood D, Simons K (2010). Science.

[19] Grant BD, Donaldson JG (2009). Nat. Rev. Mol. Cell Biol..

[20] van Deventer S, Arp AB, van Spriel AB (2021). Trends. Cell. Mol. Biol..

[21] Shorthouse D, Hedger G, Koldsø H, Sansom MS (2016). Biochimie.

[22] Liu H, Kwong B, Irvine DJ (2011). Angew. Chem..

[23] Kim HJ, Ogura S, Otabe T, Kamegawa R, Sato M, Kataoka K, Miyata K, Cent ACS (2019). Sci..

[24] Dharmaratne NU, Jouaneh TMM, Kiesewetter MK, Mathers RT (2018). Macromolecules.

[25] Lai H, Zeng D, Liu C, Zhang Q, Wang X, Chen T (2019). Biomater..

[26] Morvan MG, Lanier LL (2016). Nat. Rev. Cancer.

[27] Yang S (2019). Small.

[28] Tatsumi K (2012). Biomaterials.

[29] Vabbilisetty P, Boron M, Nie H, Ozhegov E, Sun X-L (2018). ACS Omega.

[30] Uvyn A, De Coen R, Gruijs M, Tuk CW, De Vrieze J, van Egmond M, De Geest BG (2019). Angew. Chem. Int. Ed..

[31] Ikonen E, Zhou X (2021). Dev. Cell.

[32] Kanevskiy LM, Telford WG, Sapozhnikov AM, Kovalenko EI (2013). Front. Immunol..

[33] Aqbi HF, Wallace M, Sappal S, Payne KK, Manjili MH (2018). J. Leukocyte Biol..

[34] Smyth MJ (2005). Mol. Immunol..

[35] Hwang DH, Koh P-O, Kang C, Kim E (2021). Phytomedicine.

[36] Jorgovanovic D, Song M, Wang L, Zhang Y (2020). Biomark. Res..

[37] Kim S, Li S, Gajendiran M, Jangid AK, Lee D-J, Jung H-S, Kim K (2023). Chem. Eng. J..

[38] Zanoni I, Ostuni R, Marek LR, Barresi S, Barbalat R, Barton GM, Granucci F, Kagan JC (2011). Cell.

[39] Humeau J, Bravo-San Pedro JM, Vitale I, Nunez L, Villalobos C, Kroemer G, Senovilla L (2018). Cell Calcium.

[40] Sun Y, Sukumaran P, Varma A, Derry S, Sahmoun AE, Singh BB (2014). Biochim. Biophys. Acta. Mol. Cell Res.&nbsp;.

[41] Yeh C-J, Zulueta MML, Li Y-K, Hung S-C (2020). Org. Biomol. Chem..

[42] Jin J, Krishnamachary B, Mironchik Y, Kobayashi H, Bhujwalla ZM (2016). Sci. Rep..

[43] Tsuji R, Ogata S, Mochizuki S (2022). Bioorg. Chem..

[44] Kotla NG, Bonam SR, Rasala S, Wankar J, Bohara RA, Bayry J, Rochev Y, Pandit A (2021). JCR.

[45] Bhattacharya DS, Svechkarev D, Souchek J, Hill TK, Taylor M, Natarajan A, Mohs AM (2017). J. Mater. Chem. B.

[46] Xu H, Niu M, Yuan X, Wu K, Liu A (2020). Exp. Hematol. Oncol..

[47] Misra S, Heldin P, Hascall VC, Karamanos NK, Skandalis SS, Markwald RR, Ghatak S (2011). FEBS J..

[48] Al-Othman N, Alhendi A, Ihbaisha M, Barahmeh M, Alqaraleh M, Al-Momany BZ (2020). Breast Dis..

[49] Mattheolabakis G, Milane L, Singh A, Amiji MM (2015). J. Drug Targeting..

