# Supplementary material for: Tailoring tumor-recognizable hyaluronic acid–lipid conjugates to enhance anticancer efficacies of surface-engineered natural killer cells
Source: Nano Converg. 2023 Dec 14;10:56. doi: 10.1186/s40580-023-00406-1 (PMC10721593; doi:10.1186/s40580-023-00406-1)
Supplement: Supplementary file 1 — Additional file 1: Figure S1. FTIR spectra of HA, DMPE-PEG-NH2 and synthesized HA-PEG-DMPE. [3396 cm–1 OH stretching of HA, 3269 cm–1 NH stretching of newly formed amide and DMPE-PEG- amides, 2925 cm–1 symmetric and asymmetric CH stretching of DMPE chain, 2883 and 2848 cm–1 CH stretching of both HA and DMPE, 1736 cm–1 >C=O ester bond stretching of DMPE-PEG-NH2, new broad peak at 1647 cm–1 stretching due to newly formed amide bond, 1563–1344 cm–1 CH and NH bendings of both HA and DMPE, 1280 cm–1 C-N stretching of DMPE and newly formed amide bond, 1147, 1080 and 945 cm–1 C–O–C and CO stretchings of both HA and DMPE]. Figure S2. FTIR spectra of HA, DSPE-PEG-NH2 and synthesized HA-PEG-DSPE. [3398 cm–1 OH stretching of HA, 3274 cm–1 NH stretching of newly formed amide bond and DSPE-PEG- amides, 2917 cm–1 symmetric and asymmetric CH stretching of DSPE lipid chain, 2888 and 2851 cm–1 CH stretching of both HA and DSPE lipid chain, 1736 cm–1 >C=O ester bond stretching present in DSPE-PEG, sharp peak at 1647 cm–1 stretching frequency for newly formed amide bond, 1562–1344 cm–1 CH and NH bendings of both HA and DSPE lipid chains, 1251 cm–1 C-N stretching of DSPE lipid and formed amide bond between HA and DSPE lipid, 1151, 1079, 1041 and 945 cm–1 C–O–C and CO stretchings frequencies due to both HA and DSPE lipid]. Figure S3. FTIR spectra of HA, Chol-PEG-NH2 and synthesized HA-PEG-Chol. [3411 cm–1 OH stretching of HA, 3277 cm–1 NH stretching of amides, 2945 cm–1 CH stretching of chol, 2870 cm–1 CH stretching of both HA and chol, 1731 cm–1 >C=O of chol ester, broad peak at 1645 cm–1 due to newly formed amide bond, 1563–1302 cm–1 CH bendings of both HA and chol, 1250-945 cm–1 C–O–C and CO stretchings of both chol and HA]. Table S1. Detailed IR frequencies of hyaluronic acid (HA), DMPE-PEG-NH2, and HA-PEG-DMPE biomaterial. Table S2. Detailed IR frequencies of hyaluronic acid (HA), DSPE-PEG-NH2, and HA-PEG-DSPE biomaterial. Table S3. Detailed IR frequencies of hyaluronic acid (HA), [file 40580_2023_406_MOESM1_ESM.docx]

Additional file 1

**Tailoring Tumor-recognizable Hyaluronic Acid-Lipid Conjugates to Enhance Anticancer Efficacies of Surface-engineered Natural Killer Cells**

Chae Eun Lee^a,#^, Sungjun Kim^a,#^, Hee Won Park^a^, Wonjeong Lee^a^, Ashok Kumar Jangid^a^, Yonghyun Choi^c^, Woo-Jin Jeong^b,*^, Kyobum Kim^a,*^

^a^Department of Chemical & Biochemical Engineering, Dongguk University, Seoul, Republic of Korea 04620

^b^Department of Biological Engineering, Inha University, Incheon, Republic of Korea 22212

^c^Department of Chemical Science and Engineering, Tokyo Institute of Technology, Kanagawa, Japan 226-8501

#These authors contributed equally to this work.

***Co-corresponding authors:**

**Prof. Woo-Jin Jeong, PhD**

Department of Biological Engineering, Inha University, Incheon, Republic of Korea 22212

E-mail address: wjjeong@inha.ac.kr

**Prof. Kyobum Kim, PhD**

Department of Chemical & Biochemical Engineering, Dongguk University, Seoul, Republic of Korea 04620

E-mail address: kyobum.kim@dongguk.edu

**Tailoring Tumor-recognizable Hyaluronic acid-Lipid Conjugates to Enhance Anticancer Efficacies of Surface-engineered Natural Killer Cells**

**Chae Eun Lee^a,#^, Sungjun Kim^a,#^, Hee Won Park^a^, Wonjeong Lee^a^, Ashok Kumar Jangid^a^, Woo-Jin Jeong^b,*^, Kyobum Kim^a,*^**

**Fig. S1.** FTIR spectra of HA, DMPE-PEG-NH_2_ and synthesized HA-PEG-DMPE. [3396 cm^–1^ OH stretching of HA, 3269 cm^–1^ NH stretching of newly formed amide and DMPE-PEG- amides, 2925 cm^–1^ symmetric and asymmetric CH stretching of DMPE chain, 2883 and 2848 cm^–1^ CH stretching of both HA and DMPE, 1736 cm^–1^ >C=O ester bond stretching of DMPE-PEG-NH_2_, new broad peak at 1647 cm^–1^ stretching due to newly formed amide bond, 1563–1344 cm^–1^ CH and NH bendings of both HA and DMPE, 1280 cm^–1^ C-N stretching of DMPE and newly formed amide bond, 1147, 1080 and 945 cm^–1^ C–O–C and CO stretchings of both HA and DMPE].

**Fig. S2.** FTIR spectra of HA, DSPE-PEG-NH_2_ and synthesized HA-PEG-DSPE. [3398 cm^–1^ OH stretching of HA, 3274 cm^–1^ NH stretching of newly formed amide bond and DSPE-PEG- amides, 2917 cm^–1^ symmetric and asymmetric CH stretching of DSPE lipid chain, 2888 and 2851 cm^–1^ CH stretching of both HA and DSPE lipid chain, 1736 cm^–1^ >C=O ester bond stretching present in DSPE-PEG, sharp peak at 1647 cm^–1^ stretching frequency for newly formed amide bond, 1562–1344 cm^–1^ CH and NH bendings of both HA and DSPE lipid chains, 1251 cm^–1^ C-N stretching of DSPE lipid and formed amide bond between HA and DSPE lipid, 1151, 1079, 1041 and 945 cm^–1^ C–O–C and CO stretchings frequencies due to both HA and DSPE lipid].

**Fig. S3.** FTIR spectra of HA, Chol-PEG-NH_2_ and synthesized HA-PEG-Chol. [3411 cm^–1^ OH stretching of HA, 3277 cm^–1^ NH stretching of amides, 2945 cm^–1^ CH stretching of chol, 2870 cm^–1^ CH stretching of both HA and chol, 1731 cm^–1^ >C=O of chol ester, broad peak at 1645 cm^–1^ due to newly formed amide bond, 1563–1302 cm^–1^ CH bendings of both HA and chol, 1250-945 cm^–1^ C–O–C and CO stretchings of both chol and HA].

**Table S1.** Detailed IR frequencies of hyaluronic acid (HA), DMPE-PEG-NH_2_, and HA-PEG-DMPE biomaterial.

| HA | DMPE-PEG-NH_2_ | HA-PEG-DMPE |
| --- | --- | --- |
| 3282 cm^–1^ OH stretching | 3384 cm^–1^ N–H stretching due to amine and amide bonds | 3396 cm^–1^ OH stretching of HA, 3269 cm^–1^ NH stretching of newly formed amide and DMPE-PEG-NH_2_ |
| 2890 cm^–1^CH stretching | 2925 cm^–1^, 2884-2858 cm^–1^ symmetric and asymmetric CH stretching | 2925 cm^–1^ symmetric and asymmetric CH stretching of DMPE chain, 2883 and 2848 cm^–1^ CH stretching of both HA and DMPE |
| 1602 cm^–1^ >C=O stretching of both COOH and acetyl moiety | 1737 cm^–1^ >C=O stretching of ester bond,  a small peak at 1646 cm^–1^ stretching of amide bond | 1736 cm^–1^ >C=O ester bond stretching of D DMPE-PEG-NH_2_, new broad peak at 1647 cm^–1^ stretching due to newly formed amide bond |
| 1406–1372 cm^–1^ OH bending | 1536-1359 cm^–1^ CH and NH bendings | 1563–1344 cm^–1^ CH and NH bendings of both HA and DMPE |
| 1151-947 cm^–1^ C–O–C stretching | 1342-1279 cm^–1^ C–N stretching frequencies | 1280 cm^–1^ C-N stretching of DMPE and newly formed amide bond |
|  | 1146-1060 cm^–1^ both CO and C-O-C stretching frequencies | 1147, 1080 and 945 cm^–1^ C–O–C and CO stretchings of both HA and DMPE |

**Table S2**. Detailed IR frequencies of hyaluronic acid (HA), DSPE-PEG-NH_2_, and HA-PEG-DSPE biomaterial.

| HA | DSPE-PEG-NH_2_ | HA-PEG-DSPE |
| --- | --- | --- |
| 3290 cm^–1^ OH stretching | 3398 cm^–1^ NH stretching of terminal amine and amide bond | 3398 cm^–1^ OH stretching of HA, 3274 cm^–1^ NH stretching of newly formed amide bond and DSPE-PEG-NH_2_ |
| 2890 cm^–1^CH stretching | 2916 cm^–1^, 2884-2850 cm^–1^ symmetric and asymmetric CH stretching of DSPE lipid chain | 2917 cm^–1^ symmetric and asymmetric CH stretching of DSPE lipid chain, 2888 and 2851 cm^–1^ CH stretching of both HA and DSPE lipid chain |
| 1605 cm^–1^ >C=O stretching of both COOH and acetyl moiety | 1738 cm^–1^ >C=O stretching of ester bond, a small peak about 1694 cm^–1^ stretching of amide bond present between DSPE and PEG | 1736 cm^–1^ >C=O ester bond stretching present in DSPE-PEG-NH_2_, sharp peak at 1647 cm^–1^ stretching frequency for newly formed amide bond |
| 1406–1377 cm^–1^ OH bending | 1531-1359 cm^–1^ CH bending of lipid and NH bending between lipid and PEG | 1562–1344 cm^–1^ CH and NH bendings of both HA and DSPE lipid chains |
| 1150-947 cm^–1^ C–O–C and CO stretching frequencies | 1342-1279 cm^–1^ C-N stretching of amide bonds, | 1251 cm^–1^ C-N stretching of DSPE lipid and formed amide bond between HA and DSPE lipid |
|  | 1146, 1104, 1060, and 961 cm^–1^ both CO and C-O-C stretching frequencies | 1151, 1079, 1041 and 945 cm^–1^ C–O–C and CO stretchings frequencies due to both HA and DSPE lipid |

**Table S3**. Detailed IR frequencies of hyaluronic acid (HA), Chol-PEG-NH_2_, and HA-PEG-Chol biomaterial.

| HA | Chol-PEG-NH_2_ | HA-PEG-Chol |
| --- | --- | --- |
| 3280 cm^–1^ OH stretching | 3380 cm^–1^ N–H stretching due to amine and amide bonds | 3411 cm^–1^ OH stretching of HA, 3277 cm^–1^ NH stretching of amides |
| 2885 cm^–1^CH stretching | 2946 cm^–1^ CH stretching of double bond, 2883 cm^–1^ CH stretching of alkyne both CH_2_ and CH_3_ | 2945 cm^–1^ CH stretching of Chol, 2870 cm^–1^ CH stretching of both HA and Chol |
| 1606 cm^–1^ >C=O stretching of both COOH and acetyl moiety | 1731 cm^–1^ >C=O of ester bond, 1649 cm^–1^ >C=O of amides | 1731 cm^–1^ >C=O of Chol ester, broad peak at 1645 cm^–1^ due to newly formed amide (CONH) bond |
| 1406–1376 cm^–1^OH bending | 1556-1359 cm^–1^ CH bendings | 1563–1302 cm^–1^ CH bendings of both HA and Chol |
| 1149-946 cm^–1^ C–O–C stretching | 1341-1279 cm^–1^ C-N stretching | 1250-945 cm^–1^ C–O–C and CO stretchings of both HA and Chol |
|  | 1240-1060 cm^–1^ both CO and C-O-C stretching |  |


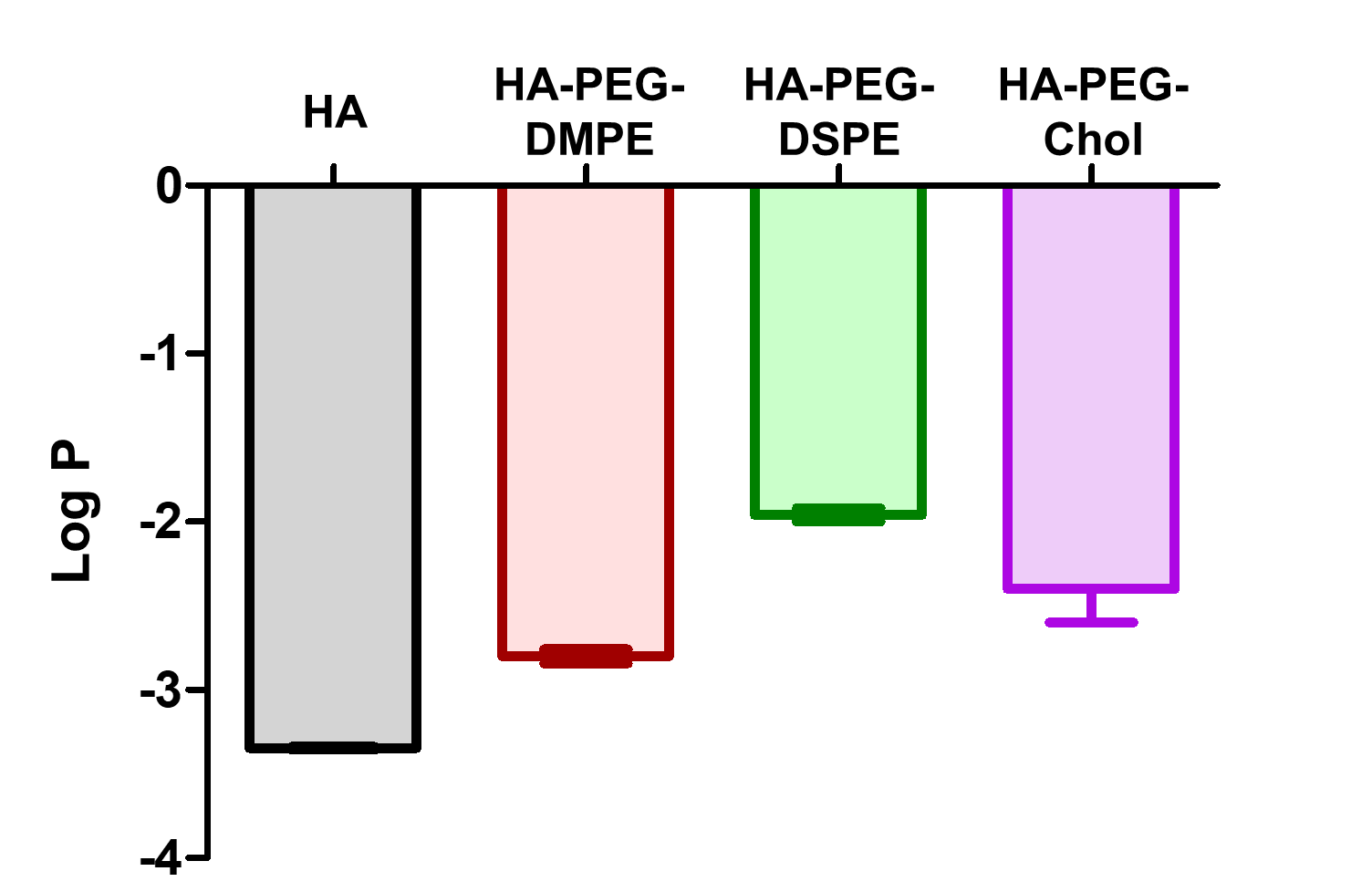


**Fig. S4.** Log P values for HA and HA-PEG-Lipid (i.e., HA-PEG-DMPE, HA-PEG-DSPE, and HA-PEG-Chol) to assess the hydrophobicity of these amphiphilic materials.
